# Professional consciousness and pride facilitate evidence‐based practice—The meaning of participating in a journal club based on clinical practice reflection

**DOI:** 10.1002/nop2.440

**Published:** 2020-02-05

**Authors:** Malene Beck, Charlotte Simonÿ, Heidi Bergenholtz, Susanne Hwiid Klausen

**Affiliations:** ^1^ Institute of Health Department of Nursing Science Aarhus University Aarhus C Denmark; ^2^ Department of Neurology Zealand University Hospital Roskilde Denmark; ^3^ Department of Physiotherapy and Occupational Therapy Slagelse Hospital Slagelse Denmark; ^4^ Institute of The Regional Health University of Southern Denmark Odense Denmark; ^5^ Section of Nursing Institute of Health Aarhus University Aarhus C Denmark; ^6^ Medical and Surgical Department Holbaek Holbaek Hospital Holbaek Denmark; ^7^ Knowledge Centre for Rehabilitation and Palliative Care (REHPA) National Institute of Public Health University of Southern Denmark Odense Denmark; ^8^ Department of Pediatrics Zealand University Hospital Roskilde Denmark

**Keywords:** nurses, nursing evidence‐based practice, research

## Abstract

**Aim:**

An evidence‐based approach should permeate clinical nursing practice, but many nurses lack confidence in applying relevant research evidence to clinical practice. Journal club participation can increase evidence‐based practice knowledge and skills while facilitating positive attitudes among participants. Thus, the aim was to describe the experience of nurses in participating in a journal club based on a curriculum derived from their practice narratives.

**Design:**

The study employed a phenomenological hermeneutical approach. Qualitative data from six focus groups with 19 nurses were interpreted in a three‐step process influenced by the French philosopher Paul Ricoeur.

**Methods:**

Influenced by narrative pedagogy and critical reflection through problem‐based learning, a journal club named *Reflexivity* was facilitated in three 2‐hourly workshops over eight weeks and evaluated together with nurses across three clinical departments.

**Results:**

Three themes are identified: (a) professional reflections are an oppressed aspect of daily nursing; (b) revealing nursing from the hidden; and (c) emerging consciousness in nursing. The study concludes that *Reflexivity* has the potential to integrate evidence‐based knowledge and increase professional consciousness by reflection on clinical questions in an evidence‐based context. Fundamental issues of nursing care are raised, and feelings of essential pride in nursing are facilitated. Thus, evidence‐based nursing practice is embarked through a journal club based on the participant's narratives.

## 1 INTRODUCTION

Despite the need and the desire among many practicing nurses to include evidence‐based knowledge in clinical nursing practice, doing so is challenging. In‐depth knowledge of methods to overcome this challenge is warranted. A structured journal club incorporating reflective practice was developed and implemented in a Danish university hospital setting. The concept is described, and findings from an interview study of the significance to staff nurses and nurse educators of participating in *Reflexivity* are described and discussed.

## 2 BACKGROUND

Patients benefit from evidence‐based nursing practice to experience meaningful health benefits and avoid suboptimal or even harmful interventions (Grol & Grimshaw, 2003). An evidence‐based approach should permeate every aspect of nursing practice and is a high priority for healthcare institutions worldwide (ibid.). Globally, it is a challenge for health systems to optimally use evidence that can reduce inappropriate use of knowledge and improve the quality of life (Straus et al., 2011). Knowledge arising from rigorous research evidence should be incorporated into care to give more useful services and improve health care and patient satisfaction. Recognition of this challenge has created interest in implementation science, the integration of knowledge translation into clinical practice (Straus et al., 2011). Journal clubs for nurses are a key strategy to facilitate evidence‐based practice (EBP) (Häggman‐Laitila, Mattila, & Melender, 2016). However, it is not clear how nurses experience participating in journal clubs and further whether participating in journal clubs may facilitate concordance between fundamental clinical experiences and research‐based evidence.

Nurses’ perceptions, attitudes and beliefs about EBP are statistically significant predictors of research uptake (Lizarondo, Grimmer‐Somers, & Kumar, 2011); it is relevant to investigate how to initiate a discussion between nurses and researchers. Although many nurses have positive attitudes and beliefs about EBP, many also find that the best research evidence is not readily available in a form that is immediately applicable in clinical practice and they lack confidence in their evidence‐based practice skills (Saunders & Vehviläinen‐Julkunen, 2016). Since journal clubs are a vital strategy to facilitate research among nurses (Fink, Thompson, & Bonnes, 2005), it is necessary to investigate how they can help ensure that the best research evidence will be easily available and applicable to clinical practice (Fink et al., 2005).

### 2.1 Aim

To investigate what it means to staff nurses to participate in structured evidence‐based reflections in a journal club setting that draws on their narratives from clinical practice.

## 3 METHODS

A qualitative study employing focus groups and a phenomenological hermeneutic approach was designed. Data analysis and interpretation were based on the philosophy of the French philosopher Paul Ricoeur (1976).

### 3.1 Design

#### 3.1.1 The concept of *Reflexivity*

**Figure 1:**
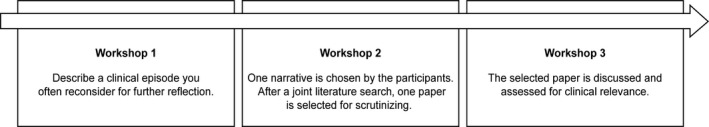
Overview of workshops in the concept of reverberation

A combination of selected elements from narrative pedagogy (Brady & Asselin, 2016) and critical reflection through problem‐based learning (Hmelo‐Sivler 2004) may motivate staff nurses to apply relevant evidence‐based knowledge to clinical practice and boost confidence in their skills. Furthermore, it is assumed that raising questions for reflection based on staff nurses’ narratives has the potential to identify fundamental issues of nursing care relevant to the participating nurses. On these considerations, the concepts of Reflexivity were developed. Figure 1 illustrates the process when participating in *Reflexivity*. Table 1 illuminates the core elements in the concept of Reflexivity.

**Table 1:** Presentation of the rationale and content of the concept

Purpose
The idea behind Reflexivity was developed and adjusted based on collaborative research on pedagogical experiences and experiences with previous journal clubs. The purpose is to cultivate professional dedication, and the engagement of participants is crucial. A level of engagement that both reflects and demands sincere dedication from participating nurses is crucial to the significance of their professional work. This includes emotional engagement, which supports positive energy and motivation. To allow genuine engagement to be in the foreground, the overall approach claims high dignity. It is crucial to meet the nurses with respect and sincere interest into their preoccupations. Therefore, the providers of the concept must hold a high engagement in both clinical nursing and evidence‐based knowledge. The concept draws upon the theory of Personalism that originates from of the French philosopher Emmanuel Mounier's philosophy of Personalism. Here, the method is used to support the professional to develop a critical consciousness that allows the individual's potential to unfold in practice (Mortensen, 2014). Dialogue is the main medium. A respectful dialogue where nurses and researchers meet and collaborate on their consciousness of nursing is necessary. Hence, the concept is both rewarding to the participating nurses and the researchers that provides the course.
Participants and providers
Participation is based on the desire to develop oneself as a nurse. The providers must have pedagogical reflections and a tentative plan that ensures participating nurses develop their critical professional consciousness. A core element of the pedagogical plan is that participants are responsible for chosen content. Reflection on one's experiences of nursing is a rewarding experience and is maintained as the foundation of the course. Participants are encouraged to write a narrative about a significant experience from their work life. This narrative is the basis of the work, and providers engage with the narrative, challenging aspects of the story and thus supporting participants in identifying one or more plots contained in the narrative. This process guides participants to reflect on the aspects of the narrative and the movement in their individual professional consciousness (Ricoeur, 1991). In this way, the curriculum of the course is a collaboration between participants and providers.
Structure
The course is held over 8 weeks. The curriculum derives from participants’ narratives that explore demanding clinical cases that they have experienced and continue to reflect on. Nurses are invited to join groups of 3–5 participants. In the sessions, participants meet in a quiet room with two providers, who have both nursing backgrounds and research education and profiles.
Session 1—Introduction to the group: two‐hour meeting
Participants meet one another and the providers and asked to share their motivation for participating. Providers introduce the purpose of the course, which is to develop a critical professional consciousness through reflections on both clinical practice and evidence. After introducing key evidence‐based practice concepts, participants are each asked to describe a demanding case from clinical practice. Providers ensure that a non‐judgemental and trusting atmosphere is established and maintained in the group. Participants are encouraged to speak freely and openly about their work as nurses and their reflections on their work. They are invited to engage with curiosity, questions, creativity, honesty and patience. To ensure transparency, providers continuously identify and discuss key words and concepts. Crucial interpretations are presented and discussed.
Self‐study—Identification of relevant issues: email correspondence within two weeks
The participants send their cases to the mentor, who poses several clinical questions for each case. Each participant assesses whether or not the clinical questions are relevant to the case.
Session 2—Selection of focus for the sessions: two‐hour meeting
The group of participants decides the case and clinical question that forms the basis for an evidence search. A search question is collaboratively formulated using PICO or SPIDER tools (Cooke, Smith, & Booth^2012^)). The group chooses one research paper for self‐study, supplemented by relevant guidelines for the study design provided by the mentor
Self‐study—Identification of relevant issues: email correspondence within two weeks
As described above
Session 3—Discussion of the nursing subject and the article: two‐hour meeting
The group appraises the quality of the evidence presented in the selected research article, discusses the selected clinical question in an evidence‐based research context and discusses implementation approaches in clinical practice.

*Reflexivity* is a structured facilitation of evidence‐based reflections, promoting active learning from nurses’ narratives from clinical practice. The concept was named *Reflexivity* to indicate that a central structure throughout the journal clubs is a reflective inherent movement based on fundamental clinical experiences and that higher level of reflections may be accomplished (by participating in the course). Reflexivity is a method of establishing a relevant discussion about core elements of nursing between facilitators and staff nurses; here, relevance is defined by nurse practitioners. The learning outcomes and curriculum were defined by nurses, and the researcher's role was to facilitate the process. To our knowledge, these pedagogical strategies have not previously been used concerning journal clubs.

### 3.2 Focus group interviews

To study the nurses’ perspectives on participating in the structured evidence‐based reflections, we used a qualitative approach consisting of focus group interviews influenced by the methods described by Halkier (2009). The interviews allowed the nurses to disclose their clinical experiences on participating in dynamic discussions with the opportunity to elaborate on this by their own concepts and language (Halkier 2009). The interviews were analysed and interpreted using a phenomenological hermeneutic approach influenced by the French philosopher Paul Ricoeur's theory on narrative and interpretation (1979, 2002). The nurses’ narratives obtained in the focus group interviews were used to elucidate their experiences of engaging in evidence‐based reflections during *Reflexivity*.

### 3.3 Participants

Nineteen staff nurses participated in six different but similar courses of *Reflexivity* between June 2017–November 2018. Participants were motivated and selected in cooperation with the departmental management team (Malterud, 2011). To obtain variation, the participant's selected differed in age, educational level, seniority and experience in nursing care (Halkier, 2009). Six focus group interviews were held after session 3 of the *Reflexivity* course. All participants consented to participate in the interviews.

### 3.4 Data collection

The focus group interviews took place at two different hospitals in three different departments: a paediatric department (seven nurses), a neurological department (six nurses) and a medical and a surgical department (six nurses). The interviews occurred between December 2017–December 2018. A semi‐structured interview guide influenced by Halkier (,^2009^, ^2010^), Morgan (1996) was used. CS and MB conducted the interviews. The interviewers strived to let the participants disclose their experiences with the concept of *Reflexivity* based on the course in open and dynamic discussions and acted as a moderator to encourage discussion and elaboration among participants about their experiences and perspectives in a dynamic and trusting setting (Halkier, 2009, 2010; Morgan, 1996). The interviews were held in a quiet meeting room and lasted between 45–70 min. Initial impressions were immediately written down, and digitally recorded conversations were transcribed verbatim.

### 3.5 Data analysis and interpretation

Ricoeur (1976) describes the phenomenological–hermeneutic approach as a spiral following the text and its movements from “*sense*” to “*reference*” or from “*what the text says to what the text talks about*” (Ricoeur, 1976, p. 87–88). By continually moving between the whole text and parts of the text, the authors achieved what Ricoeur calls a “sophisticated understanding” (Ricoeur, 1976). This sophisticated understanding gave a new in‐depth and nuanced understanding of what it meant to the nurses to participate in *Reflexivity*. The process of analysis and interpretation included a dialectical movement among a naïve reading, structural analysis, critical interpretation and discussion (Dreyer & Pedersen, 2009; Pedersen, 2005). *Naïve reading*: MB and CS read and reread the text several times. This process made it possible to grasp an initial overall understanding of the content. *Structural analysis*: The content of the text was organized into units of importance. This process was compared with the naïve reading and referred to by units of meaning, consisting of quotes from the interviews. Thus, it became possible to explain and understand the text and to identify themes. To achieve the most trustworthy interpretation of the text, all authors contributed to this process. *Critical interpretation and discussion*: The themes were interpreted and discussed with the involvement of the philosophy of the Austrian pedagogical philosopher Martin Buber (1937) along with existing research.

### 3.6 Ethical considerations

In this study, the ethical guidelines of the Helsinki Declaration were followed, and the nurses participated voluntarily and were guaranteed that their statements were not recognizable. The participants were informed verbally and in writing about the purpose of the study and their anonymity and confidentiality throughout the study. The Danish Data Protection Agency approved of the study (REG‐ 209–2017), and their guidelines on the storage of data were followed. The regional ethics committee in Region Zealand, Denmark, stated that approval was not needed because the study was not biomedical. Furthermore, it was stated before the interviews started that the confidentiality also applied to participants to establish a trustful situation where the respondent felt secure that their statements were not taken outside the room.

### 3.7 FINDINGS

#### 3.7.1 Naïve reading

Based on the naïve reading, the concept of *Reflexivity* seems a stepping stone for working with evidence‐based reflections on nursing based on staff nurses’ narratives about their clinical practice. “Evidence‐based reflections” are defined as reverbing self‐addressed clinical questions in a research evidence‐based context. Experiences with reflecting on self‐addressed clinical issues deriving from clinical practice and based on their professional engagement facilitated professional pride and made it possible to ask critical questions to the nursing profession, as it was presented by the participants themselves. In the material, it was repeatedly implied that reflection on one's professionalism and working with evidence‐based practice was not a part of staff nurses' job responsibilities. It was indicated that being critically reflective was not permeated into daily clinical nursing practice and that this practice was perceived as something reserved for a few employees. *Reflexivity* made the participants realize that reverbing a clinical question is a method of establishing learning close to clinical practice and that Reflexivity potentially facilitated an “educational journey” to reclaim time for nurses to reflect in clinical settings. It seems that participating in *Reflexivity* was a revelation to staff nurses. Throughout the course, the nurses experienced a growing sense of nursing professionalism that gave rise to a wish to include evidence‐based knowledge to their care practices.

### 3.8 Structural analysis

#### 3.8.1 Professional reflection is an oppressed aspect in daily nursing practice

During the interviews, it became clear that the staff nurses were longing for colleagues to listen to their perspectives from clinical practice. Clearly, staff nurses were discouraged, struggled with busy work conditions and felt they had to adjust their nursing intentions down. Clinical settings were described as chaotic, which made the staff nurses reflect on their practice as distant, foreign and hidden. One nurse gave an example by saying: *We are bouncing out there, but we never get the chance to talk about that thing called “nursin*g” (F4). Listening to the staff nurses’ narratives in the concept of *Reflexivity* was named as a remarkably valuable educational tool. Thus, it shed light on staff nurses’ perception that structured reflections on nursing were reserved for academic nurses only. One staff nurse gave an example of how evidence‐based reflection was a distant activity with no relevant connection to the clinical context:

They have hired some nurses who will find something scientific and try to teach you, but I do not listen. It is like “you will HAVE TO listen.” Yes, but I need to sit, eat and relax. It's (the teaching) always at either the morning coffee or lunch break. I want to sit with my phone and merely be allowed to have 10 minutes by myself. I do not need to listen to anything. The clinical situation is vulnerable. There are no resources at all and the staff is so… they do not need someone to tell about the latest results within one area. You need a break. (F1)

The staff nurses identified how their experiences with reflections and evidence‐based practice were described as impersonal and somewhat mechanical. Hence, evidence‐based reflections should be implemented with the same efficiency as the morning routine with meetings and breakfast. The staff nurses did not consider themselves as capable of engaging in reflective educational activities in the framework of daily clinical work. Yet, the staff nurses were eager to have room for reflection on clinical practice. A staff nurse elaborated on addressing clinical questions during working hours:

… we are just not encouraged to do it. To me, at least, it is on our initiative that we do so. That’s too bad. On a busy day, it often ends up with fundamental considerations: “Have the patients eaten? Have the patients slept? Have the patients been to the toilet?” So we rarely move beyond this and have in‐depth reflections or thoughts on why we do things. (F4)

The staff nurses’ experiences with addressing and reflecting on clinical questions were distant and foreign from their everyday clinical practice, which resulted in rather tragicomic statements when asked during the interviews. This statement is representative of when some of the nurses were asked to elaborate on whether their staff meetings dealt with scientific results or communicated results from new research. The staff nurses laughed loudly and said:

Sorry, I do not laugh at you (talking to the interviewer). I mostly laugh at ourselves. At one point, we had a person… She presented some research at our 10 o’clock meeting. “Try to read the paper and then we could talk about it,” she said. It's challenging to be ready for these meetings. Then you have a little breakfast and someone's professional thoughts. It's so stressful. I do not learn anything at these meetings. (F4)

Timing and a sense of *when* reflection could occur and *how* setting a framework for *which* environment reflection should occur were lacking. For example, the staff nurses described how communicating, educating and reflecting on nursing care was based on articles being handed out, *stuck into your hand in the doorway* (F1) or placed in the nurses’ desk drawer, meaning that nurses did not feel ownership. Thus, there was no relation between research and daily nursing practice. Experiencing morning meetings where science was *flushed down with coffee and white bread* made the staff nurses miss ownership to deal with reflections on their own clinical stories; hence, no community to identify a substantial aspect of nursing was addressed. One staff nurse illustrates the premise for evidence‐based practice in clinical practice by saying:

I think the evidence is a word that is very “heavy.” It has some strength behind it. It's a small word, but it contains a lot. You get very far with the evidence and it paves the way. But … I also want to say … that in practice, the word saves a little. It is not in focus, which I might well miss. (F3)

#### 3.8.2 Revealing nursing from the hidden

*Reflexivity* is perceived as a method for staff nurses to become attached to some of the clinical or organizational issues they experienced daily and serves as an opportunity to reflect on clinical practice based on their narratives:

Reflexivity has been a break to me in some aspects. It has been helpful to talk about the problems we experience. One thing is that the management is aware, but to unfold it here… during Reflexivity and also to do some literature search related to it has been excellent. You can empty your backpack, in a way. (F1)

The staff nurses connected to professional thoughts and evidence‐based practice that contrasted favourably with how they experienced everyday practice. Thus, the concept of *Reflexivity* was appealing to the nurses and encouraged them to address nursing care with a more positive and creative attitude. One staff nurse illustrates this notion by describing her thoughts of *Reflexivity*:

Reflexivity had a beautiful sound and it seemed as though I would use it (the course) for something. Reflexivity sounds so harmless. Although it was a search for literature or was about research, it was a positive word. Reflexivity tasted like… (making chewing sounds while thinking). I got curious (F3). Often, I feel I am stuck when I am at work. I do the same thing every single day, e.g., rounds, care. Here, (during Reflexivity), I can just put that (routine thinking) away and breathe. (F1)

The staff nurses considered the concept of *Reflexivity* as meaningful because it was a way to be present as a professional. The nurses described how the concept allowed them to address professional thoughts and ideas they had hidden away. Therefore, the nurses described *Reflexivity* as an appreciated activity that facilitated inspiration and gave life to professional actions for a brief moment. Words, such as “breathing” (F1), “creating” (F5), “boosting” (F2) or “dynamic” (F4), describe how the concept of *Reflexivity* was rewarding to staff nurses. Their choice of words reflected essential elements about their perspectives on clinical practice. This was related to the experience of a space, where one could dwell and let (professional) thoughts wander off. A staff nurse described it like this:

From the beginning, I thought o.k. This is akind of mindfulness. Reflexivity has been valuable to me. It has been a way to be thoughtful. Reflective. It has been a way for me to look into literature, but at the same time, it has, not at a high level, connected everyday life to how we experience the situation out there (in clinical practice). (F5)

*Reflexivity* seemed meaningful as it mirrored clinical practice and gave an invitation to feel ownership of certain professional areas. One staff nurse said: “*To me, has represented many different aspects, but it is primarily an eye‐opening event to the fact that we as nurses can take more ownership concerning patients and their needs”* (F4). Having the opportunity to reflect and immerse themselves as staff nurses in daily experiences providing care to patients and collaborating with one another was a new and calming aspect to the staff nurses’ (work) life that put things into useful and meaningful perspectives. Thus, the voice of the staff nurses was raised when the nurses engaged with explaining, understanding and theorizing clinical issues:

It has been excellent with examples from everyday life; however still connected to something bigger. Here, there has been peace and quietness for doing that. They created that room without it being too tight (F6). I feel that having participated in Reflexivity has meant that there has been room for my thoughts; that… I have had a positive gain, that's not something I can necessarily go out and use in clinical settings right away, but it has given me something personally. I have been boosted. (F4)

#### 3.8.3 Emerging consciousness in nursing

Focusing on nursing and the everyday life that staff nurses experience in the hospital was a positive driver for solving issues in clinical practice. Participating in *Reflexivity* meant that staff nurses presented a case that they often recalled, and one problem was formulated as a research question based on this experience. This process provided the staff nurses with a sense of trust in themselves about their professional skills and a feeling of ownership of a specific problem they could address:

It has been nice that it was merely our reality or experiences that were used in the cases and formed the basis for searching literature. Then, you have the feeling that it is possible. That you can be in a dilemma and then find some relevant information that can be used. I also think that the ownership becomes much stronger if it is a situation that you have been in. (F5)

The material covered that staff nurses' understanding related to evidence‐based knowledge of nursing. Gaining evidence‐based knowledge among novice nurses was a meaningful activity because they were familiar with this methodology from their education and they referred to their education not being “used” or “asked for” when working as a nurse in the hospital. Being able to address academic competencies in, for example, searching for literature is a method to address the emerging curiosity they had felt during their education. However, in many ways, this curiosity was oppressed by everyday clinical practice:

Recently, when I graduated and had read a lot of articles, I considered myself a skilled professional. And now… well. So, when I got the opportunity to sit for myself, write a case and become aware of how many aspects each situation includes, it was an eye‐opener. You just run around with so many things every day that you have to decide what is stored in your mind and what is not. However, focusing on the reflections is very important for you to be able to finish and do well with each patient. If you have too many “loose ends,” it can be very messy. (F6)

By analysing and interpreting the interview data, we identified that the concept of *Reflexivity* was an eye‐level method of addressing evidence‐based practice for staff nurses. The concept was different from the traditional methods of reflecting on nursing and gave a new experience in evidence‐based practice for staff nurses. Here, the academic process of searching for literature, selecting a scientific article and asking critical questions about the content, was based on their cases and reflections on nursing care. So, in addition to learning how to search databases, the staff nurses also addressed and systematically worked with reflections on core elements of their nursing practices. Being a part of such a process made the nurses understand that their cases addressed nursing in the way that it was carried out in real life:

Reflexivity has meant that I am more confident about my own intuition. It helps to know that others had been occupied with the same and that enables you to process situations and putting others word on something that you have also experienced. (F5)

When the staff nurses were asked to suggest improvements to *Reflexivity*, they replied that they wished to have more time to do literature search and more homework. At the same time, they stated that this competence was not demanded in clinical practice or at the management level. The staff nurses experienced that they could become relevant collaborators for other groups (especially the physicians) if they could demonstrate that they could handle evidence‐based knowledge as a part of their professional practice. Thus, *Reflexivity* was not just a matter of learning to search for literature but also a method of advocating for nursing and therefore establishing an equal conversation across professional disciplines:

We have been confirmed that nursing is as important as medical treatment. Of course, medical treatment is vital, but I think that you should use things like Reflexivity to build up a professional backbone. Even though this is 2018, it is still so that as a nurse, if you are doing nursing, then you put it aside to help a doctor who says “can you, could you come and help me with this.” There, we have to stand up for nursing and say, “I'm doing something and that is just as important for the patient”. (F4)

Obtaining knowledge based on a case selected by the staff nurses and reading articles gave the staff nurses a sense of security in their professionalism. Feeling comfortable with nursing by gaining new knowledge meant that staff nurses achieved a state of calmness. Being able to feel calm by obtaining new knowledge related to cases the staff nurses themselves had selected was an important finding because it was in contrast to the chaos they otherwise described in clinical practice. A staff nurse formulated this during an interview by saying: *In Reflexivity, we can lean on knowledge and theorists. That provides peace and security (F6).* Achieving peace and security in relation to one's profession meant that the nurses gained ownership of particular areas associated with patients, which made it easier to accept their everyday clinical life:

Reflexivity has, to me been a reminder that if you take the time to search for literature, then you also find the academic arguments to your dilemmas. That leads to higher job satisfaction (F5). Also, that you know that you can discuss something in a professional language with your colleagues … and then know that you have the back‐up from the researchers upstairs. (F3)

*Reflexivity* gave an opportunity for staff nurses to get to know each other across the nursing discipline. For example, how to justify development and research in nursing, as well as the description of different vacant positions, together with the possibilities of collaboration between nurses and researchers was discussed. Thus, the participants experienced that they could get (academic) help if they needed it. A nurse describes how this relationship made a bodily impression on her:

Now we know that they (MB, HB, & SHK) can be helpful. We have become more visible to each other. We know we coexist. It is like we have touched each other differently now and that makes us perhaps use or “draw” on one other. (F2)

### 3.9 Comprehensive understanding and discussion

Based on our findings, it can be argued that when staff nurses are supported in raising study questions based on their clinical practice narratives, the tone of discussions changes from casual to professional. By reformulating nurses’ reflections into professional language, opportunities to search for and apply research‐ and evidence‐based knowledge increase. Our study shows that journal clubs based on narratives enhance staff nurses’ beliefs about the value of evidence‐based research. Addressing self‐selected clinical questions in a journal club context boosted staff nurses’ confidence about addressing problems in their practice and reflecting on clinical issues in an evidence‐based framework.

Nurses in our study described a lack of interest in a working environment where they discuss care related to their actions and reflect on each other's thoughts and considerations. Therefore, the concept of *Reflexivity* is at risk of being perceived as something that must be directly transferable to and applied in clinical practice, in addition to its value for nurses’ professionalism on a daily basis. However, participants perceived their personal reflections as having little or no value before they experienced the positive effect of reflection at a more abstract level on their nursing practice. Our study gives new knowledge by illustrating that the process of participating in *Reflexivity* gives a meaningful discussion in professional language between researchers and staff nurses that is centred on participants' narratives. A shared interest in core nursing subjects is clarified and identified. Accordingly, staff nurses, as well as their academic colleagues, gained a sense of professional pride and enhanced their commitments to professional reflection, which contrasted favourably with their descriptions of clinical settings.

A recent systematic review of journal clubs for nurses found that researchers often have a central role in selecting articles (Häggman‐Laitila et al., 2016). This finding is in contrast to our study that, consistent with earlier research, emphasizes that other clinical activities will be prioritized unless personal motivation to reflect on professionalism during journal clubs is mobilized (Squires, Estabrooks, Gustavsson, & Wallin, 2011). Our study documents that it is possible to conduct a journal club where participants experience being a part of the process of investigating essential issues in nursing. A model where staff nurses are an integral part of the process of formulating nursing narratives, choosing topics and searching for literature compares favourably with a more traditional method of conducting journal clubs (Dall’Oglio et al., 2018; Häggman‐Laitila et al., 2016; Patel et al., 2011). We also found that, in daily clinical practice, no tradition exists of reflecting on one's nursing practice by searching for and reading research articles. Academic reflection in *Reflexivity* exemplifies an innovative use of problem‐based learning; the curriculum is not defined initially, and few requirements for participants' commitment other than motivation exist. The voluntary nature of participation is voluntary, and the deep involvement of participants in developing the course and process is essential to whether they find the reflections valuable and further integrate research‐based knowledge into their practice (Fink et al., 2005).

Based on a comprehensive understanding of our findings, it can be argued that, although human learning is a lifelong journey, an innovative journal club is an excellent supplement to nursing practice. Learning and reflection through reciprocal discussion and the acquisition of new knowledge enhance nurses’ beliefs about the value of the evidence‐based practice. Formulating one's professional identity happens through discussion with others about recognizable and essential narratives from clinical practice. This perspective is based on the philosophy of Martin Buber (1878–1965).

In his first work, *I and Thou* (1937), Buber focuses on the significance of the relationship between people. According to Buber, the relationship between people defines who man (one) is. The entirety of existence life consists of relationships because one is always in relationships. Even when alone, one's thoughts emerge in the context of relationships. Buber emphasizes that humans are both relational and dialogic beings. Dialogue is more than just a conversation. In Greek, dialogue means “through words” and connotes the intention of speaking through words. In dialogue, one seeks and expresses intention through words and the intention in dialogue is most important in interaction with others.

Taking this philosophical framework into account, the concept *Reflexivity* is based on a pedagogical view where working with evidence‐based practice can be viewed as a balance of giving and receiving and guiding a dialogue to achieve a common good for patients in clinical practice.

Giving refers here to the efforts of researchers to teach, guide and provide nurses with space and a framework, enabling the collective commitment of nurses to reflection and learning about evidence to become the central driving force. It is important that the context where reflections are solicited and discussed is a safe space where nurses find the motivation and courage to develop professionally. It also means that the researchers give of themselves and their professionalism. For example, one's research and experiences can be used in several ways to help participating nurses learn about the many facets of nursing research. Concerning *Reflexivity*, giving also refers to the need for the concept to have a clear framework to support and encourage nurses during the course.

Receiving in this context means being receptive in the dialogue with participating nurses, so all participants’ contributions are balanced in volume and status. We learn alongside participating nurses in dialogue and reflections on practice. It is crucial to be open and listen to the dialogue when working with evidence‐based practice and establishing journal clubs, allowing both nurses and researchers to integrate the thoughts and perspectives that arose during discussions into their daily work. Based on our findings, we believe that a prerequisite for successful evidence‐based nursing practice is enhancing nurses’ expectations of evidence‐based practice and allowing their stories to be actively incorporated into the process. Our study also revealed that researchers could be very influenced by nurses' narratives when designing, developing and evaluating new research studies in clinical practice. Buber (1937) supports the perspective that dialogue provides nurses with acknowledgement of who they are as professionals. Thus, *Reflexivity* illuminates nascent aspects of nursing identity that were previously unformed. Throughout the process, pride and feelings of safety concerning discussion with other professionals are nourished and flourished.

### 3.10 Strengths and limitations

This study was conducted only in one setting. In terms of generalization, it would have been preferable to study nurse's perspectives in more contexts (to gain nuanced insight into how nurses articulated the opportunity to engage with evidence‐based practice based on their own narratives). Furthermore, we collected data when *Reflexivity* was in progress and did not explore how participants actually applied the knowledge they obtained in the process, for example literature search in clinical practice. Nevertheless, our findings give new insight into the phenomena of learning and reflecting on clinical practice being a nurse practitioner. This study can be considered as pilot study of an innovative usage of problem‐based learning in a journal club setting, to supplement nursing practice and improve patient satisfaction.

Study participants were selected based on interest in participating they expressed to departmental managers and they worked in different clinical areas. Nurses who were selected for participation by departmental management may have experienced the course differently. Also, this study included a relatively small number of participants. Selecting participants based on other factors, such as an application, interview or informal conversations with nursing staff, might add variation to the recruitment of participants and allow journal clubs to emerge in new ways (Malterud, 2011).

## 4 CONCLUSION

The concept of *Reflexivity* is an educational experience for nurses that fosters their reflection on their actions in a clinical context. Supporting nurses in the professional development of evidence‐based practice based on their narratives strengthens their engagement in meaningful reflection on daily nursing actions. Reflexivity renews professional concepts, theories and methods. Nursing is an individual and a collective form of professional activity with distinct theoretical concepts and methods that need to become activated daily to achieve practice excellence.

*Reflexivity* solicited participants’ often very personal descriptions of demanding experiences that were sometimes written in emotional and informal terms and reframed them in professional language. Familiar theoretical concepts and methods became revitalized and could be applied in discussions, as personal descriptions nurtured the integration of meaningful, evidence‐based knowledge into clinical practice.

## 5 RELEVANCE TO CLINICAL PRACTICE

The findings of this study create awareness of how participating in a journal club based on personal narratives from clinical practice boosted participants’ confidence and pride in nursing. Reframing clinical questions in an evidence‐based context is a way of creating a community of awareness in the nursing profession between staff nurses and nurse researchers. An inspiring dialogue is initiated between colleagues with different educational perspectives and skills at addressing challenges in daily nursing practice. Improvements in clinical nursing practice through future collaboration are likely to result. It is notable that the entire process was completed over eight weeks and involved a minimal time commitment from participants.

## CONFLICT OF INTEREST

None declared.

## AUTHOR CONTRIBUTIONS

M.B, C.S, H.B and SHK: Study design, Data collection analysis and Manuscript preparation.

## ETHICS APPROVAL

The study was approved by the Danish Data Supervisory Committee by Region Zeeland (REG‐209–2017).
